# Editorial: Exploiting new methods to study microglia in healthy and diseased retina

**DOI:** 10.3389/fimmu.2023.1177065

**Published:** 2023-03-16

**Authors:** Anton Lennikov, Kin-Sang Cho

**Affiliations:** Schepens Eye Research Institute of Massachusetts Eye and Ear, Department of Ophthalmology, Harvard Medical School, Boston, MA, United States

**Keywords:** editorial, microglia, new methods, advances, review, retina

## Introduction

Microglia are a type of immune cells found in the retina that play a crucial role in maintaining retinal homeostasis, protecting against injury, and responding to disease (1). In a healthy retina, microglia help to clear cellular debris, maintain synapses and support neuronal survival (2). In a diseased retina, however, microglia can activate and contribute to retinal inflammation and neurodegeneration (Rashid et al.).

The study of microglia in the healthy and diseased retina is rapidly evolving. This Research Topic brings together a collection of contributions that showcase new and innovative methods for investigating these important immune cells. In this special issue, there are two review papers. Targeting microglia to treat degenerative eye diseases review focuses on the role of microglia in degenerative eye disorders. It highlights various strategies to target eye microglia to treat rare and common ocular diseases (Wang et al.). These strategies include depleting microglia with chemicals or radiation, reprogramming microglia using homeostatic signals or other small molecules, and inhibiting the downstream effects of microglia, such as by blocking cytokine activity or phagocytosis. The paper discusses the use of genetic and pharmacological methods for depleting microglia in animal models and provides details on various small molecules, such as CSF1R inhibitors (3). Modulation of microglia function through modulation of receptor-ligand interaction CX3CL1, CD200, TGF-β, and others that have been tested to reprogram microglia. The review also highlights the potential use of IGF-1, TUDCA, and GLP-1R agonists to modulate the activity of microglia in the eye. Finally, the paper identifies areas of future research needed to fully exploit the therapeutic value of microglia in eye diseases.

Continuing with the problem of microglia contribution to neuroinflammation and its impact on retinal ganglion cell survival, the second review paper focuses on the role of resident retinal microglia in facilitating Wallerian degeneration and subsequent axon regeneration after optic nerve crush (ONC) injury as well as their production of pro-inflammatory cytokines, chemokines, and reactive oxygen species that have neurotoxic effects on retinal ganglion cells (RGCs) (Au et al.). The paper highlights the poor phagocytic capacity of resident retinal microglia in clearing cellular and myelin debris, which is a major contributor to the formation of growth-inhibitory myelin debris and a glial scar that inhibit RGC axon regeneration and visual function recovery in patients with traumatic optic neuropathy. The review also discusses the impact of intraocular inflammation and chronic neuroinflammation on secondary tissue damage and visual function recovery after ONC. The potential therapeutic strategies target the controlled activation of retinal microglia to foster neuroprotection and nerve repair.

In the original research contributions, the manuscript by Wolf et al. presents novel data on important questions on how different the retinal and brain microglia are as compared to these results with peripheral monocytes. The authors isolated human retinal microglia from enucleated eyes and compared their transcriptional profile with whole retinal tissue, human brain microglia, classical, intermediate, non-classical monocytes, and murine retinal microglia. The findings showed that human retinal microglia exhibited a high degree of similarity to their counterparts in the brain but with several enriched genes compared to other cell types. The study also identified several species-specific genes in human and murine retinal microglia, which should be considered when using mouse models to study retinal microglia biology and its projection to human diseases.

In the more clinical research contribution, Zeng et al. presented findings on the clinical features of macrophage-like cells (MLCs) in retinal vein occlusion (RVO) using en-face optical coherence tomography (OCT). These infiltrating cells are likely to consist of retinal microglia and inflitrating macrophages (MLCs). The research involved 36 patients with RVO, and the MLCs were binarized and quantified using a semiautomated method. The results showed that the morphology of MLCs in RVO eyes appeared larger and plumper, and the density of MLCs was significantly higher in the affected region than in the unaffected region. The study suggests that the increased density and changes in morphology characterized by OCT may indicate generalized activation and aggregation of MLCs in RVO. The role of MLCs in the clinical consequences of retinal diseases is a growing interest. The study presents a novel *in vivo* imaging method to visualize individual MLCs on the inner limiting membrane of the human retina using clinical en face OCT.

The final manuscript in the collection is the original research investigating retinal hyper-reflecting foci (HRF) in the OCT images in the retina of multiple sclerosis (MS) patients that may represent clusters of activated and proliferating microglia (Puthenparampil et al.). In this, the authors investigated the association of HRF with cerebrospinal fluid (CSF) cytokines and MRI parameters in relapsing-remitting MS (RRMS) patients at clinical onset. They found that HRF count in the ganglion cell layer (GCL) was associated with certain cytokines and that CSF concentrations of certain cytokines were also associated with global cortical thickness. HRF count in the inner nuclear layer (INL) correlated with different cytokines, and the CXCL-13/CXCL-2 ratio was strongly associated with HRF count and cortical lesion volume. The authors suggest that the association of HRF with cytokines confirms their microglial origin and indicates they may be useful as markers of activated microglia. OCT with a single linear scan through the macula proved an effective method for *in vivo* evaluation of activated microglia in the retina.

In conclusion, studying microglia in the retina is an area of active research. The contributions in this Research Topic highlight new and innovative methods for investigating these immune cells. The review papers shed light on the potential of targeting microglia to treat degenerative eye diseases and to foster neuroprotection and nerve repair. The original research contributions provided valuable insights into the molecular profile of human retinal microglia, the clinical features of macrophage-like cells in retinal vein occlusion, and the use of retinal hyper-reflecting foci as markers of activated microglia in MS. Overall, the findings presented in this special issue enhance our understanding of microglia biology in the retina and pave the way for new therapeutic approaches for retinal diseases.

## Author contributions

The editorial was written by AL and K-SC and revised by AL, both authors reviewed and accepted the final version of the manuscript.
